# A New Nano-Platform of Erythromycin Combined with Ag Nano-Particle ZnO Nano-Structure against Methicillin-Resistant *Staphylococcus aureus*

**DOI:** 10.3390/pharmaceutics12090841

**Published:** 2020-09-02

**Authors:** Atanu Naskar, Sohee Lee, Yunhee Lee, Semi Kim, Kwang-sun Kim

**Affiliations:** 1 Department of Chemistry and Chemistry Institute for Functional Materials, Pusan National University, Busan 46241, Korea; atanunaskar@pusan.ac.kr (A.N.); kin5497170@pusan.ac.kr (S.L.); 2 Immunotherapy Research Center, Korea Research Institute of Bioscience and Biotechnology, Daejeon 34141, Korea; yunhee@kribb.re.kr (Y.L.); semikim@kribb.re.kr (S.K.)

**Keywords:** low-temperature synthesis, ZnO nano-structure, antibacterial activity, antibiotic resistance, biocompatibility

## Abstract

Nano-particles have been combined with antibiotics in recent studies to overcome multidrug-resistant bacteria. Here, we synthesized a nano-material in which Ag nano-particles were assembled with a ZnO nano-structure to form an Ag-ZnO (AZO) nano-composite at low temperature. This material was combined with erythromycin (Ery), an antibiotic effective towards gram-positive bacteria, using three different approaches (AZO + Ery (AZE) [centrifuged (AZE1), used separately after 1-h gap (AZE2), without centrifugation (AZE3)]) to prepare a nano-antibiotic against clinical isolates of methicillin-resistant *Staphylococcus aureus* (MRSA). X-ray diffraction analysis and transmission electron microscopy confirmed the presence of Ag nano-particles and ZnO nano-structure. The elemental and chemical state of the elements present in the AZO nano-composite were assessed by X-ray photoelectron spectroscopy. The antibacterial activity of AZE samples against both *Escherichia coli* and *S. aureus* strains including MRSA was evaluated in antibacterial and morphological analyses. The AZE3 sample showed greater antibacterial activity than the other samples and was comparable to erythromycin. AZE3 was ~20-fold less prone to developing bacterial resistance following multiple exposures to bacteria compared to erythromycin alone. The AZE3 nano-composite showed good biocompatibility with 293 human embryonic kidney cells. Our newly synthesized nano-platform antibiotics may be useful against multidrug-resistant gram-positive bacteria.

## 1. Introduction

In recent years, the term multidrug-resistant (MDR) has become synonymous with bacterial infections, which have shown increased resistance to available antibiotics, requiring immediate attention [1]. The real threat of the MDR can be realized by estimating that MDR could cause 10 million annual deaths by 2050, surpassing cancer [1]. In addition, this threat might be exacerbated through the heavy use of antibiotics in the developing COVID-19 pandemic to keep patients away from secondary bacterial infections. To overcome above issues, researchers have attempted to identify new classes of antibiotics, chemically modify existing drugs, and use alternatives to current antibiotics, and other methods. In particular, nano-materials have emerged as promising alternatives to current antibiotics to treat infections caused by MDR pathogens. The distinctive physiochemical properties of nano-materials enable their use in numerous biomedical applications such as bacterial killing, drug delivery, bioimaging, and anticancer treatments [2]. In particular, nano-materials can exhibit antibacterial activity either alone [3] or in combination with conventional antibiotics to enhance their efficiency [4].

Because of their antibacterial activities, various metal and metal oxide nano-particles such as ZnO, CuO, TiO_2_, SiO_2_, MgO, Cao, Ag, Au, and Cu offer effective solutions for treating infections by MDR pathogens [5]. ZnO has been widely used by researchers because of its antibacterial activity and distinctive physiochemical properties such as high surface area-to-volume ratio [6,7]. The Food and Drug Administration has already recognized ZnO as a “GRAS” (generally recognized as safe) substance (FDA, 21CFR182.8991, USA) [8]. Moreover, ZnO preferentially targets multiple bacterial pathways rather than using the single target approach of antibiotics [9]. This strategy makes it difficult for bacteria to develop resistance against ZnO. Silver nano-particles (Ag NPs), another metal oxide nano-particle, have been extensively used because of their antibacterial activity [10]. From ancient times, Ag has been used as an antibacterial and antifungal agent. However, the importance of Ag NPs has recently increased because Ag NPs are less likely to result in bacterial resistance and can be used in multi-dimensional approaches to exert antibacterial activity. Moreover, Ag NPs are used in commercial (textiles, biopolymer, and coating-based products) and medical (burn wound treatment and dental work) approaches [11].

Materials prepared by combining NPs and antibiotics, known as nano-antibiotics, have recently been developed to enhance the activity of antibiotics without causing the acquisition of resistance by bacteria [12]. As potential nano-antibiotic platforms, researchers have combined Ag NPs with conventional antibiotics to enhance antibacterial activity [4]. Similarly, ZnO has been used with antibiotics to potentiate their synergistic actions [13]. However, combinations of Ag, ZnO, and antibiotics have not been reported. Generally, antibiotics exert antibacterial activity either by directly interacting with bacterial components or by inhibiting their biosynthesis and degradation processes. Among the currently used antibiotics, erythromycin is on the List of Essential Medicines published by World Health Organization (Geneva, Switzerland) and is considered to be the safest and most effective medicine [14]. Thus, erythromycin is widely used against both gram-positive and gram-negative bacteria to inhibit bacterial protein synthesis [15]. However, bacteria can resist the activity of erythromycin by reducing their interactions with erythromycin, increasing the risk of bacterial infections that negatively affect human health. Specifically, *Staphylococcus aureus* (*S. aureus*) has rapidly developed resistance to erythromycin [16,17]. In contrast, NPs target multiple bacterial pathways compared rather than the single targeting used by antibiotics, and thus bacteria are less prone to become resistant to NPs [18]. Therefore, combining Ag-ZnO NPs with erythromycin may show similar antibacterial activity while reducing drug resistance. This strategy may enable the application of currently ineffective therapies against bacterial infections to be repurposed without time- and money-consuming development of a new generation of antibiotics.

Therefore, the aim of this study is to develop a new promising nano-platform of erythromycin, which selectively inhibit the growth of *S. aureus* cells with inhibiting a rapid increase of bacterial resistance by use of erythromycin itself. To this end, we prepared a new nano-antibiotic platform composed of Ag NP assembled with a ZnO nano-structure and combined with erythromycin (AZE) to potentiate both the specific activity against *S. aureus* and its MDR strains [(methicillin-resistant *S. aureus* (MRSA)), and the reduction of drug resistance caused by erythromycin. A ZnO nano-structure was synthesized through low-temperature solution synthesis followed by assembly with Ag NPs.

## 2. Materials and Methods

### 2.1. Synthesis of ZnO (ZO) Nano-Structure

A simple low-temperature precipitation process was used to synthesize ZnO (ZO). Briefly, 0.2 M aqueous solution of Zn(NO_3_)_2_·6H_2_O, (98%, Sigma-Aldrich, St. Louis, MO, USA) was prepared in a beaker with 50 mL of deionized water (DW) before continuous stirring for 15 min at room temperature. In another beaker, 1.6 M aqueous solution of sodium hydroxide (NaOH, 95%, Junsei, Tokyo, Japan) was mixed with 50 mL of DW. Then, the NaOH solution was added to the Zn(NO_3_)_2_ reaction mixture by dropwise with continuous stirring. After adding the base, a white precipitate can be seen at the bottom of beaker. The solution was stirred at 80 °C for 6 h and then transferred to an ice bath to stop the reaction. The white precipitate was collected by centrifugation. DW and ethanol were used several times to wash the product followed by drying in an air oven at 60 °C overnight.

### 2.2. Synthesis of Ag-ZO (AZO)

To obtain the Ag-ZO (AZO) nano-composite, 200 mg of synthesized ZO was dispersed in 20 mL of DW and ultrasonicated for 10 min. This solution was mixed with 20.0 mL of aqueous 0.05 M AgNO_3_ (≥99.9%, Sigma-Aldrich) with continuous stirring, after which 2.0 g polyvinylpyrrolidone (PVP-40000, Sigma-Aldrich) was added and stirred continuously for 1 h. Next, 200 µL of NaBH_4_ (0.1 M) solution was added to the mixture slowly by vigorous stirring, followed by stirring for 1 h at room temperature to form AZO. Centrifugation process was used to collect the solid materials before washing with DW and ethanol. At last, the samples were kept in an air oven for drying at 60 °C overnight.

### 2.3. Preparation of Ag-ZO-Erythromycin (AZE)

Three different techniques were applied to prepare AZE samples for antibacterial analysis. In the first technique, synthesized AZO (50 mg) and erythromycin (50 mg) were mixed in 50 mL sterilized DW and stirred overnight. The product was centrifuged and washed with DW before drying in an air oven at 60 °C overnight. This combination of AZO and erythromycin was named AZE1. In the second technique, the combinational product was named AZE2. During agar well diffusion, 10 µL of AZO (1 mg/mL) was initially added. After 1 h, 10 µL of erythromycin (1 mg/mL) was added, followed by the incubation of plates for 24 h at 37 °C. The third technique was similar to the first technique; AZO and erythromycin were mixed in the same weight ratio and stirred overnight. The product, named AZE3, was used directly to assess antibacterial activity.

### 2.4. Characterization

#### 2.4.1. Material Properties

X-ray diffractometer (D8 Advance with DAVINCI design X-ray diffraction unit, Bruker, Billerica, MA, USA) with a nickel-filtered Cu Kα radiation source (λ = 1.5406 Å) was used for X-ray diffraction (XRD) study to evaluate the structures of ZO and AZO. The 2θ range of 20–80° was used to collect the diffraction patterns. Furthermore, the microstructure of a representative sample of AZO was evaluated by transmission electron microscopy (TEM; Bruker Nano GmbH, Berlin, Germany) with the help of carbon-coated 300 mesh Cu grids for holding the samples. Scanning electron microscopy (SEM) analysis was performed using a VEGA3 (TESCAN, Fuveau, France). Additionally, an Axis Supra Scanning X-ray photoelectron spectroscopy (XPS) microprobe surface analysis system (Kratos Analytical, Manchester, UK) was used to assess the binding energy and chemical state of elements in a representative AZO sample by scanning from 200 to 1200 eV. The C 1s peak position at 284.5 eV was used as the binding energy reference.

#### 2.4.2. Preparation of Bacterial Cells

Antibacterial activity was evaluated as described previously [6] using BBL^TM^ Mueller-Hinton Broth (MHB, BD Biosciences, Franklin Lakes, NJ, USA) to grow the bacterial strains purchased from American Type Culture Collection (ATCC), Manassas, VA, USA: *Escherichia coli* (ATCC 25922), *S. aureus* (ATCC 25923), and different MRSA clinical isolates. The MRSA strains were purchased from Culture Collection of Antimicrobial Resistant Microbes (CCARM, Seoul, Korea; www.ccarm.or.kr) and validated by two individual experiments [19]. Initially, the MHB medium was inoculated with single bacterial colonies and incubated at 37 °C overnight. The cultivated cells were used within 30 min for the agar well diffusion assay (Section 2.4.3) to assess the antibacterial activity of the NPs (ZO, AZO, and AZE). In another method, cells were prepared by suspending colonies in DW. The cells were diluted to an optical density of 0.5 McFarland turbidity standard using Sensititre^TM^ Nephelometer (Thermo Fisher Scientific, Waltham, MA, USA) to determine the minimum inhibitory concentration (MIC) (Section 2.4.4) and characterize the cell morphology (Section 2.4.5).

#### 2.4.3. Agar Well Diffusion Assay

The antibacterial activities of ZO, AZO, and AZE against *E. coli*, *S. aureus*, and clinical isolates of MRSA strains (MRSA1 to 8) were evaluated by the well-known agar well diffusion assays. Initially, 500 µL of cultured bacterial cells was mixed with 25 mL of MHB-agar and poured into sterile petri dishes (ϕ = 90 mm) for solidification. In preliminary experiments, four holes, each 6 mm in diameter, were aseptically punched through the surface with a sterile plastic rod. Next, 20 µL of ZO or AZO (5 mg/mL), erythromycin (5 mg/mL, Sigma-Aldrich), or DW was dropped into the holes on the agar plate with bacterial cells as the experimental group, positive control, and negative control, respectively. The plates were incubated for 24 h at 37 °C, and the zone of inhibition (ZOI) produced by the materials was measured with a ruler. After assessing the initial results, similar procedures were performed to prepare MHB-agar petri dishes. Next, seven holes (each 6 mm in diameter) were aseptically punched and 20 µL (1 mg/mL) of (i) ZO, (ii) AZO, (iii) AZE1, (iv) AZE2, (v) AZE3 (vi) erythromycin, and (vii) DW were added. At the end of the experiment, antibacterial activities were assessed after measuring the diameter of the ZOI around the wells by using a transparent ruler.

#### 2.4.4. Minimum Inhibitory Concentration (MIC) of Antibacterial Activity

The bacteria used for this experiment were diluted in MHB at a ratio of 1/1000 in MHB. Samples of ZO, AZO, and AZE3 (5 mg/mL each) were prepared with DW by serial dilution to achieve concentrations of 1.56 to 200 μg/mL. Then, 10 μL of each diluted sample was inoculated to 90 μL of the targeted bacterial medium. The bacterial cells were incubated by shaking at 500 rpm for 16 h at 37 ℃, after which the MIC was determined.

#### 2.4.5. Morphological Characterization of Bacteria

To determine the effect of AZE3 against the same bacterium except for *E. coli*, the cells were prepared as described in Section 2.4.2. AZE3 (5 mg/mL) was added to the cell suspension to final concentrations of 25 and 100 µg/mL for *S. aureus* and MRSA strains, respectively. AZE3 (5 mg/mL) and erythromycin (5 mg/mL) were used at final concentrations of 25 and 0.125 (or 0.25) µg/mL, respectively. Each sample was incubated at 37 °C with a vigorous shaking for overnight. The bacterial cells were then pelleted at 12,000 rpm for 1 min by centrifugation. With 500 µL of phosphate-buffered saline (pH 7) containing 2% formaldehyde and 1% glutaraldehyde, the pellets were resuspended and then centrifuged again for 5 min. The resulting pellets were washed twice with 1 mL of DW and resuspended in the same volume of DW. To prepare SEM image analyses, a 5 µL of aliquots were collected from the suspension and deposited on a silicon wafer (5 × 5 mm in size, Namkang Hi-Tech Co., Ltd., Seongnam, Korea) and dried at room temperature. The dried wafer was examined by SEM using a VEGA3 (TESCAN, Fuveau, France), a versatile tungsten thermionic emission SEM system, according to the manufacturer’s instructions.

#### 2.4.6. Drug Resistance Study

To induce drug resistance, *S. aureus* cells (10^6^ CFU/mL) were incubated with AZE3 (50 µg/mL) or erythromycin (50 µg/mL) and diluted accordingly for eight passages. The antibacterial activity of AZE3 or erythromycin against each passage of *S. aureus* cells was measured and compared to determine the efficacy of the AZE3 sample.

#### 2.4.7. Cell Viability Study (Water Soluble Tetrazolium Salt, WST Assay)

293 [HEK-293] (ATCC^®^ CRL-1573™) (human embryonic kidney cells) were purchased from ATCC (Manassas, VA, USA) and maintained in RPMI1640 with 10% of fetal bovine serum at 37 °C in 5% CO_2_. A colorimetric WST assay (Ez-Cytox; Dogenbio, Seoul, Korea) was performed to evaluate the cell viability in the synthesized samples. The cells were seeded into 96-well plates at a density of 4000 cells/well and incubated for 24 h. The cells were further incubated for 24 or 48 h in the presence of ZO, AZO, or AZE3 samples at concentrations of 10 to 50 µg/mL in 0.1% dimethyl sulfoxide. The cells were then incubated with WST reagent (one-tenth of the medium volume), after which a spectrophotometric microplate reader (BMG LABTECH GmbH, Ortenber, Germany) was used to determine the amount of formazan dye formed by measuring the absorbance at 450 nm.

## 3. Results and Discussion

### 3.1. Material Properties

#### 3.1.1. X-ray Diffraction (XRD) Study

**Figure 1:**
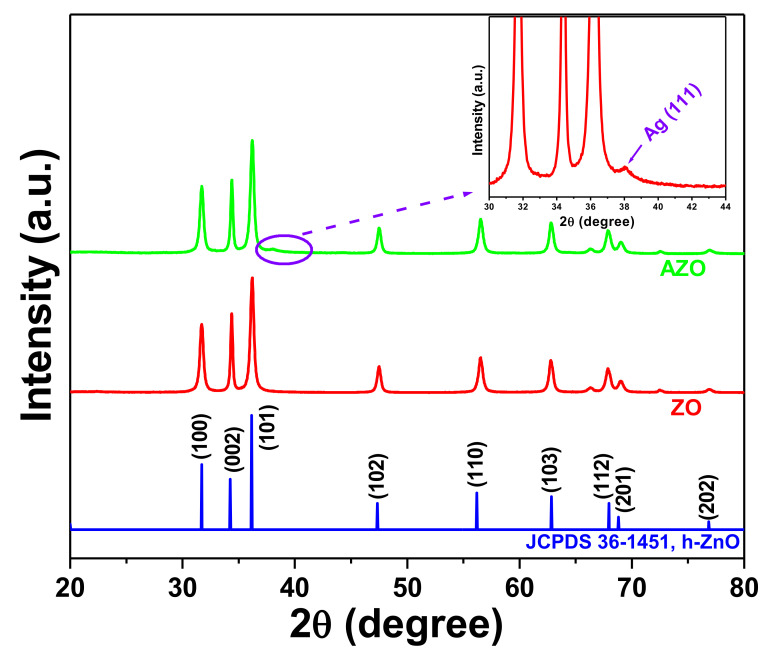
X-ray diffraction patterns of ZO and AZO samples. Inset shows the enlarged spectra (marked portion) of the AZO sample.

XRD of the synthesized samples ZO and AZO was performed to evaluate the crystalline phase of the samples (Figure 1). The XRD peaks of the as-synthesized ZO and AZO observed at 31.71°, 34.38°, 36.23°, 47.50°, 56.55°, 62.86°, 67.93°, 68.99°, and 76.97° were indexed to the diffraction planes (100), (002), (101), (102), (110), (103), (112), (201), and (202), respectively, which were consistent with hexagonal ZO (JCPDS 36-1451) [18]. Moreover, the additional peak (inset, Figure 1) observed at ~38.1° for the AZO sample corresponded to the crystal planes of cubic Ag (JCPDS 04–0783) [20]. These results confirmed the successful formation of the AZO sample.

#### 3.1.2. Morphology and Microstructure Study

**Figure 2:**
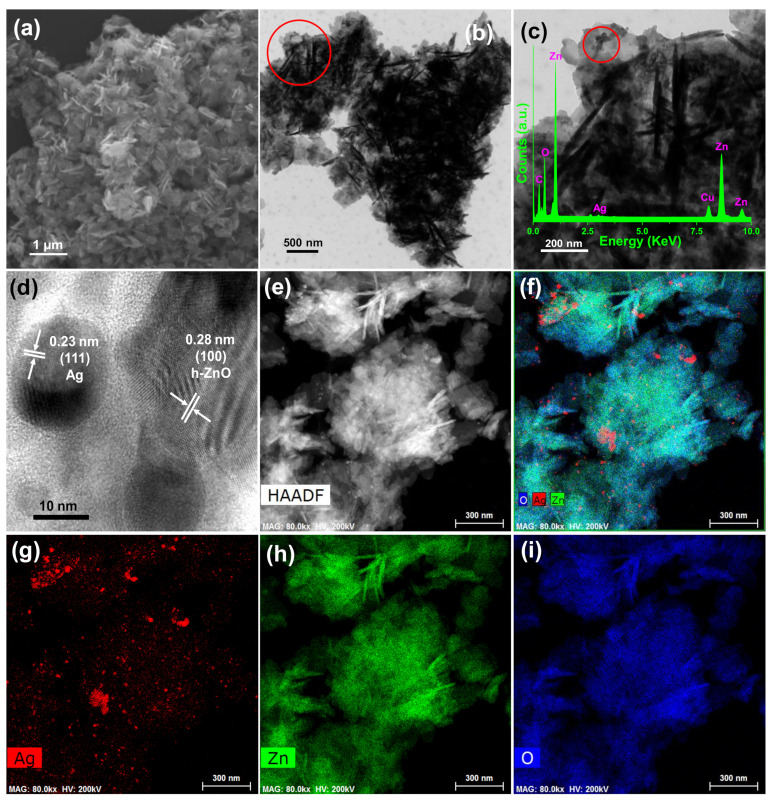
(**a**) FESEM image of as-prepared ZO sample. (**b**,**c**) Transmission electron microscopy (TEM) images of AZO sample. (**c**) Enlarged TEM image of AZO sample from the marked portion of (**b**) with (**c** inset) TEM-energy-dispersive X-ray spectrum, (**d**) high-resolution TEM image of the AZO sample from the marked portion of (**c**), High-angle annular dark-field (HAADF) image (e), and elemental mappings of (**f**) composite, (**g**) Ag, (**h**) Zn, and (**i**) O.

Figure 2a shows the field emission scanning electron microscope (FESEM) image of the as-synthesized ZO sample, clearly revealing formation the ZO nano-structure. Similarly, Figure 2b,c show TEM images of the AZO sample, which also support ZO nano-structure formation. Notably, Figure 2c shows the marked region in Figure 2b. Moreover, the high-resolution TEM image of the AZO sample (Figure 2d, from the marked portion in Figure 2c) confirmed the presence of Ag and ZO NPs, as the distinct lattice fringes with an interplanar distance of 0.23 and 0.28 nm correspond to the (111) plane of Ag and (100) plane of ZO [20], respectively. Thus, the TEM and high-resolution TEM images of the AZO sample support the XRD results (Figure 1) and confirm successful formation of AZO. Furthermore, the inset of Figure 2c shows the energy-dispersive X-ray spectral analysis of the AZO sample nano-composite, where the existence of Zn and O supported the formation of ZO nano-particles. Similarly, the formation of Ag nano-particles was supported by the presence of Ag. Additionally, the use of carbon-coated Cu grid for TEM measurements demonstrates the existence of C and Cu observed in the TEM-energy-dispersive X-ray spectrum. Elemental mapping images of a representative AZO sample exhibited good distributions of Ag (Figure 2g), Zn (Figure 2h), and O (Figure 2i). Thus, successful formation of the AZO sample was confirmed by TEM (Figure 2) and XRD (Figure 1).

#### 3.1.3. X-ray Photoelectron Spectroscopy (XPS) Results

**Figure 3:**
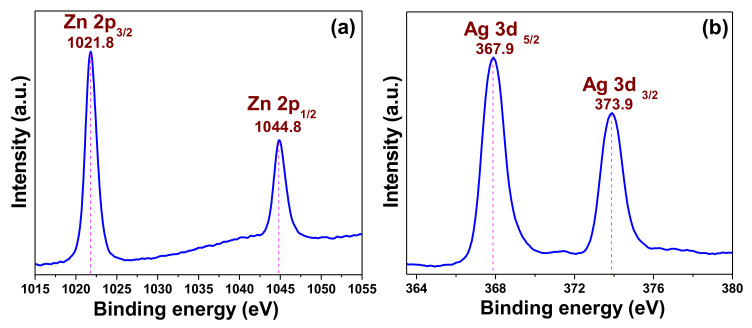
X-ray photoelectron spectroscopy binding energy spectra of AZO (**a**) Zn *2p* and (**b**) Ag *3d* core levels.

The elemental and chemical state of the elements from the representative sample AZO were evaluated by XPS. The corresponding results are shown in Figure 3. The XPS results are in corroboration with the XRD (Figure 1) and TEM-energy-dispersive X-ray spectrum (inset of Figure 2c) confirming the elements present in the sample. The results revealed the binding energy signals of Zn *2p* (Figure 3a), and Ag *3d* (Figure 3b). Two strong binding energy signals observed at 1021.8 and 1044.8 eV in Figure 3a were assigned to the binding energies of Zn *2p*_3/2_ and Zn *2p*_1/2_, respectively [20]. Additionally, the calculated energy difference between the Zn *2p*_3/2_ and Zn *2p*_1/2_ binding energy levels, which was ~23.0 eV, confirmed the presence of zinc as Zn^2+^ in the AZO sample. This result also supports the formation of ZnO in nano-composite. The two peaks at ~367.9 and ~373.9 eV in Figure 3b were respectively assigned to the binding energy signals of Ag *3d_5/2_* and Ag *3d_3/2_*, confirming the formation of metallic Ag in the AZO sample [20]. Moreover, the energies of these two peaks are comparatively lower than bulk Ag(368.2 eV, Ag *3d_5/2_*, 374.2 eV, Ag *3d_3/2_*) [21]. The reason behind this peak shifting to lower binding energies can be ascribed to the electrons transfer from metal Ag to ZnO indicating strong interaction between Ag and ZnO, confirming the formation Ag-ZnO nano-composite. Additionally, the energy difference between the two peaks of Ag *3d* (~6.0 eV) is an additional evidence of the presence of metallic Ag in the nano-composite [21]. Therefore, the XPS results support those of XRD (Figure 1) data and confirm the successful formation of the AZO nano-composite.

### 3.2. Antibacterial Activity

#### 3.2.1. Zone of Inhibition (ZOI)

**Figure 4:**
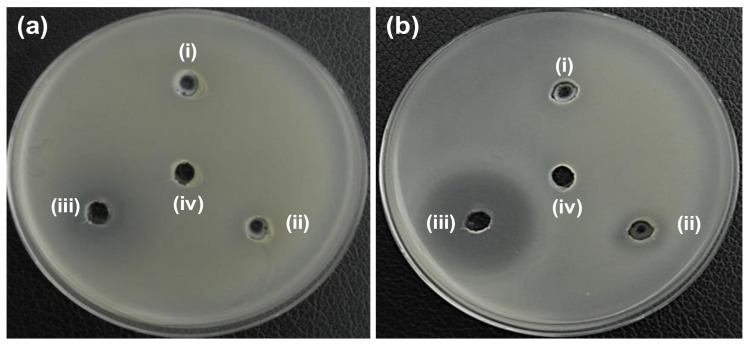
Determination of zone of inhibition (ZOI) for ZO and AZO samples against (**a**) *E. coli* and (**b**) *S. aureus*. (i), (ii), (iii), and (iv) represents ZO, AZO, erythromycin (Ery) and deionized water (DW), respectively. Diameter of ZOI measured by ruler is shown in Table 1 (average from *n* = 3).

**Figure 5:**
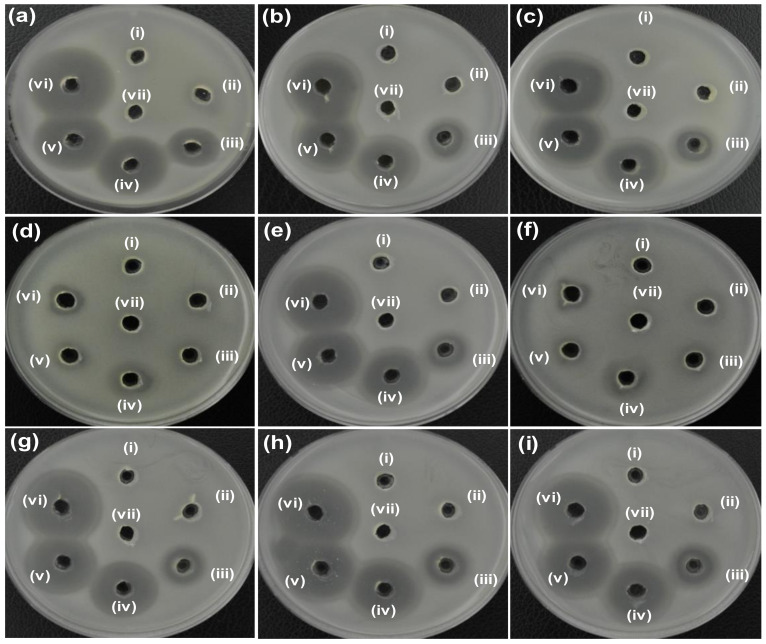
Zone of inhibition (ZOI) of ZO samples against (**a**) *S. aureus*, and (**b**–**i**) MRSA1-MRSA8. (i), (ii), (iii), (iv), (v), (vi), and (vii) represents ZO, AZO, AZE1, AZE2, AZE3, erythromycin (Ery), and deionized water (DW), respectively in all figures. Diameter of ZOI is also displayed in Table 2 (average from *n* = 3).

The antibacterial activity of ZO, AZO, and AZE samples, and their strain specificity was evaluated by the agar well diffusion method. First, 20 µL (5 mg/mL) of synthesized samples (ZO and AZO) were loaded into agar plates containing both *E. coli* (Figure 4a) and *S. aureus* (Figure 4b) strains and incubated for 24 h at 37 °C to analyze the initial antibacterial effectiveness of the synthesized nano-materials; the ZOI was measured for individual samples (Table 1). The ZO and AZO samples were ineffective against gram-negative bacterial cells of *E. coli* (Figure 4a), as no inhibition zone was formed. However, the AZO sample showed initial antibacterial activity against *S. aureus* (Figure 4b) with a 14-mm of ZOI diameter. Therefore, the AZO sample and combination of AZE was further used against MDR strains of *S. aureus* (MRSA1–8) to evaluate antibacterial activity. The results are shown in Figure 5 and the measured diameter of the ZOI is shown in Table 2. As described above, 20 µL of synthesized samples (ZO and AZO) were loaded into the agar plates but at a lower concentration (1 mg/mL). The result clearly demonstrated that the AZE3 sample was comparatively more effective against all MRSA strains (except for MRSA3 and MRSA5) compared to AZE1 and AZE2. Therefore, AZE3 was further evaluated for its antibacterial activity. This combinatorial innovative strategy of nano-material with antibiotics was effective against MRSA strains and can be used as a nano-antibiotic platform against MDR gram-positive bacterial cells.

#### 3.2.2. MIC

The MIC values (Table 3) of the ZO, AZO, and AZE3 samples were measured to confirm the antibacterial effects of the AZE3 sample against a standard strain (*S. aureus* [ATCC25923]) and MRSA isolates (MRSA1–8).

The MIC values in Table 3 clearly show that the AZE3 sample was effectively inhibited the growth of *S. aureus* and MRSA strains. The MIC values for all bacteria were 1.56–12.5 µg/mL. This antibacterial activity demonstrates the successful formation of AZO with erythromycin and their synergistic activity.

#### 3.2.3. Morphological Characterization of Bacteria

**Figure 6:**
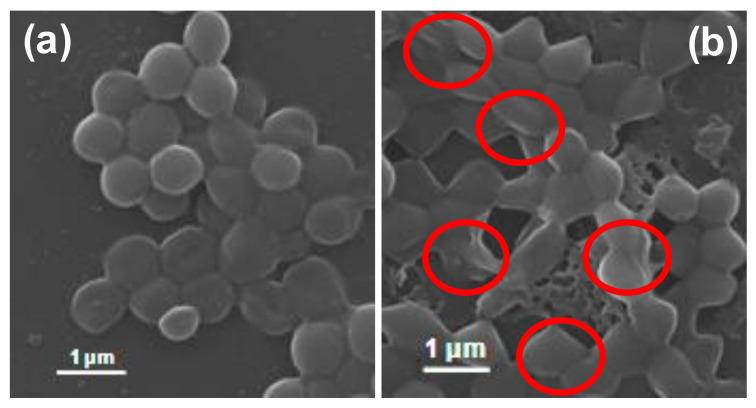
Scanning electron microscopy (SEM) images of *S. aureus* cells. *S. aureus* cells of (**a**) untreated and (**b**) treated with AZE3. Red circles indicate membrane regions of *S. aureus* damaged by AZE3.

**Figure 7:**
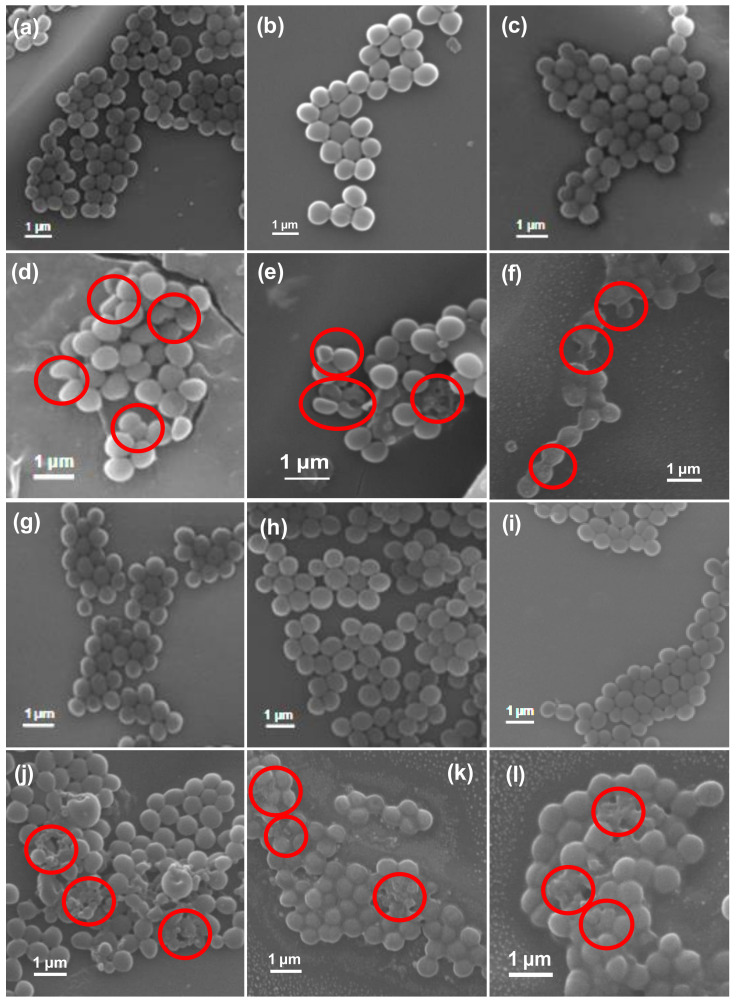
Scanning electron microscopy (SEM) images of bacterial cells. Samples of untreated MRSA1 (**a**), MRSA2 (**b**), MRSA4 (**c**), MRSA6 (**g**), MRSA7 (**h**), MRSA8 (**i**), and treated MRSA1 (**d**), MRSA2 (**e**), MRSA4 (**f**), MRSA6 (**j**), MRSA7 (**k**), and MRSA8 (**l**) with AZE3. Red circles indicate areas of cell membrane disruption.

The antibacterial efficiency of ZO samples was also evaluated by observing morphological changes in the standard and MDR strains of *S. aureus* (standard, MRSA1, MRSA2, MRSA4, MRSA6, MRSA7, and MRSA8) before and after exposure to the AZE3 sample. Figure 6 and Figure 7 show the SEM images of standard and MDR strains of *S. aureus* either treated or untreated with nano-materials. Figure 6a shows the untreated *S. aureus* cells, which clearly had a smooth and intact surface. In contrast, extensive membrane damage was observed for *S. aureus* treated with AZE3 (Figure 6b), confirming the effects of AZE3. Similar types of morphology were detected for MRSA strains with either untreated groups (MRSA1, Figure 7a; MRSA2, Figure 7b; MRSA4, Figure 7c; MRSA6, Figure 7g; MRSA7, Figure 7h; MRSA8, Figure 7i), which had a smooth and undamaged surface, and groups treated with AZE3 (MRSA1, Figure 7d; MRSA2, Figure 7e; MRSA4, Figure 7f; MRSA6, Figure 7j; MRSA7, Figure 7k; MRSA8, Figure 7l), which showed some morphological changes such as membrane damage. This activity was corroborated by the ZOI (Figure 5), showing that the AZE3 sample was more effective than other samples for inhibiting the growth of MRSA strains. Therefore, the combination of nano-particles (AZO) with antibiotics (erythromycin) synergistically killed MDR strains of bacteria cells such as MRSA. The interaction between the nano-particles and cell wall caused enhanced membrane permeability, allowing entry of AZE3 into the cell. Finally, erythromycin caused a damage on the cell, resulting the cell death. This also explains why the AZE3 sample is more effective than AZO. Thus, the effectiveness of the AZE3 sample was demonstrated by SEM micrographs against MRSA strains.

#### 3.2.4. Drug Resistance Study

**Figure 8:**
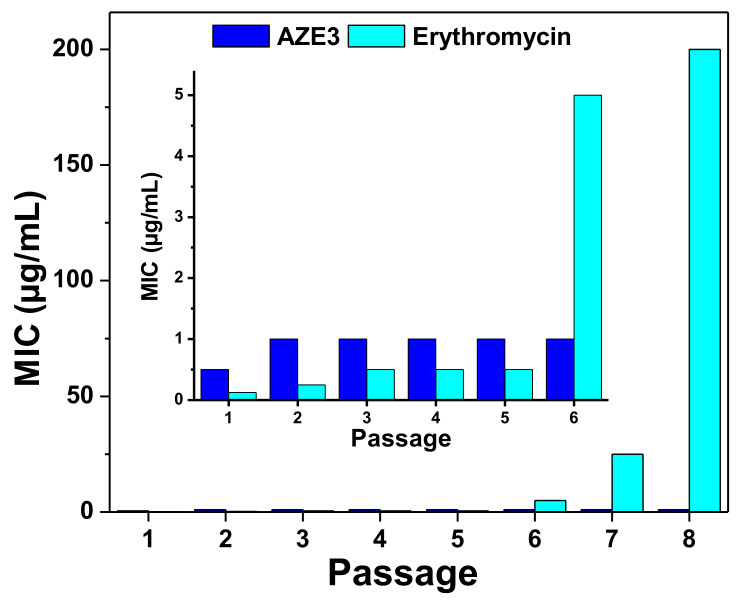
Development of drug resistance. Antibacterial profiles of AZE3 and erythromycin against *S. aureus* cells after multiple treatments (*n* = 8) at sub-lethal concentrations. Inset shows the enlarged portion form passage 1–6.

In general, antibiotics exert their antibacterial effects by interacting with bacterial components such as the cell wall, nucleic acids, or proteins and inhibit their biosynthesis. Similarly, erythromycin inhibits bacterial growth by preventing protein synthesis in bacteria [15]. Notably, bacteria can reduce their interactions with antibiotics to result in drug resistance [22]. However, the antibacterial mechanism of NPs [6,7] or, in this case, AZO NPs mainly involves membrane disruption of bacteria which occurs independently of direct interactions with bacterial components. Therefore, AZO NP may overcome resistance to antibiotics. Additionally, combining AZO NP with erythromycin antibiotics was used to form a unique nano-composite (AZE3) which merges the antibacterial mechanism of AZO NP (i.e., bacterial membrane disruption) and protein synthesis inhibition mechanism of erythromycin. Thus, this prepared AZE3 sample can efficiently inhibit the bacterial growth and shows a lower risk of drug resistance. To verify this hypothesis, *S. aureus* cells were treated with AZE3 or erythromycin at sub-lethal concentrations for eight passages, and the antibacterial activity at different passages was compared (Figure 8). The results clearly demonstrate that multiple exposures of *S. aureus* bacteria to erythromycin slowly resulted in drug resistance. In comparison, the AZE3 sample still showed antibacterial activity even after eight passages. Thus, combining AZO NP with erythromycin, i.e., AZE3, not only resulted in high antibacterial activity but also showed a lower risk of drug resistance. Therefore, the prepared AZE3 sample is a promising nano-platform of erythromycin.

### 3.3. In Vitro Cytotoxicity

**Figure 9:**
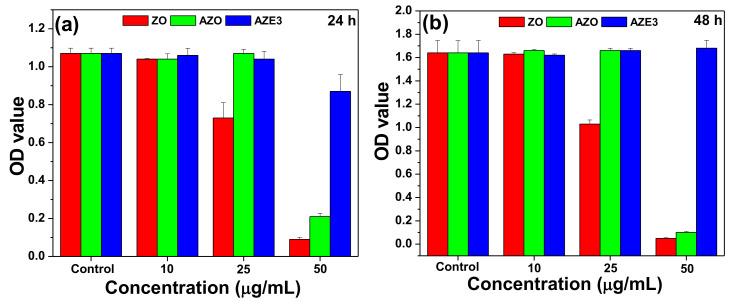
Cell viability from in vitro cellular cytotoxicity, WST assay of 293 cells (human embryonic kidney cells) for ZO, AZO, and AZE3 samples with various concentrations for (**a**) 24 h and (**b**) 48 h. The error bars represent the ±standard deviation (SD) (*p* < 0.05).

The cell viability was examined by assessing in vitro cellular cytotoxicity in a WST assay of 293 cells with varying concentrations of ZO, AZO, and AZE3; the results are shown in Figure 9. The average of triplicate measurements is shown in each bar graph. The figure clearly demonstrates that the AZE3 nano-composite had high biocompatibility. Therefore, the AZE3 nano-composite is biocompatible with human cells but toxic to bacterial cells and has potential antibacterial applications.

## 4. Conclusions

In this study, we synthesized a ZnO nano-structure which was assembled with Ag NPs to prepare an Ag-ZO (AZO) nano-composite in a low-temperature solution synthesis process. The synthesis method of the ZnO nano-structure was simple, fast, and reproducible. The material properties of the samples were characterized by XRD, TEM, and XPS. The AZO sample combined with erythromycin inhibited the growth of MRSA clinical isolates and showed a lower risk of drug resistance than antibiotics alone. Moreover, the synthesized nano-composite showed high cell viability. Therefore, this cost-effective synthesized nano-composite can be used in different biomedical applications including as an antibacterial agent.

## Figures and Tables

**Table 1:** Zone of inhibition (ZOI) diameter of ZO and AZO samples against (a) *E. coli* and (b) *S. aureus* was measured from *n* = 3 and representative data are shown.

Bacteria Cells	Zone of Inhibition (mm)			
	(i) ZO	(ii) AZO	(iv) Ery	(v) DW
(a) *E. coli* ATCC25922	N.D.	N.D.	17	N.D.
(b) *S. aureus* ATCC25923	N.D.	14	30	N.D.

N.D. indicates that the zone of inhibition was not detected. Erythromycin (Ery) was used as a control. DW: Deionized water.

**Table 2:** Zone of inhibition (ZOI) diameter of ZO and AZE against (a) *S. aureus* and (b–i) MRSA1–8 was measured from *n* = 3 and representative data are shown.

Bacteria Cells	Zone of Inhibition (mm)						
	(i) ZO	(ii) AZO	(iii) AZE1	(iv) AZE2	(v) AZE3	(vi) Ery	(vii) DW
(a) *S. aureus* ATCC25923	N.D.	12	20	28	28	32	N.D.
(b) MRSA1	N.D.	N.D.	21	26	28	30	N.D.
(c) MRSA2	N.D.	N.D.	20	27	27	30	N.D.
(d) MRSA3	N.D.	N.D.	N.D.	19	12	13	N.D.
(e) MRSA4	N.D.	N.D.	19	27	28	31	N.D.
(f) MRSA5	N.D.	N.D.	N.D.	18	13	11	N.D.
(g) MRSA6	N.D.	N.D.	15	25	26	29	N.D.
(h) MRSA7	N.D.	N.D.	19	27	27	30	N.D.
(i) MRSA8	N.D.	N.D.	14	28	29	32	N.D.

N.D. indicates that the zone of inhibition was not detected. Erythromycin (Ery) was used as a control. DW: Deionized water.

**Table 3:** Minimum inhibitory concentration (MIC) of ZO samples against (a) *S. aureus* and (b–i) MRSA1 to 8. Data shown are representative from *n* = 3.

Bacterial Cells	Minimum Inhibitory Concentration (µg/mL)		
	ZO	AZO	AZE3
(a) *S. aureus* ATCC25923	200	100	1.56
(b) MRSA1	>200	200	6.25
(c) MRSA2	>200	>200	6.25
(d) MRSA3	>200	>200	>200
(e) MRSA4	>200	>200	6.25
(f) MRSA5	>200	>200	>200
(g) MRSA6	>200	>200	12.5
(h) MRSA7	>200	>200	6.25
(i) MRSA8	>200	>200	6.25
